# Modulation of redox homeostasis: A strategy to overcome cancer drug resistance

**DOI:** 10.3389/fphar.2023.1156538

**Published:** 2023-03-22

**Authors:** Yang Li, Xiaoyue Zhang, Zhihan Wang, Bowen Li, Huili Zhu

**Affiliations:** ^1^ State Key Laboratory of Biotherapy and Cancer Center, West China School of Basic Medical Sciences and Forensic Medicine, West China Hospital, and Collaborative Innovation Center for Biotherapy, Sichuan University, Chengdu, China; ^2^ Key Laboratory of Birth Defects and Related Diseases of Women and Children of Ministry of Education, Department of Reproductive Medicine, West China Second University Hospital of Sichuan University, Chengdu, China

**Keywords:** redox homeostasis, drug resistance, cancer therapy, reactive oxygen species, redox signaling

## Abstract

Cancer treatment is hampered by resistance to conventional therapeutic strategies, including chemotherapy, immunotherapy, and targeted therapy. Redox homeostasis manipulation is one of the most effective innovative treatment techniques for overcoming drug resistance. Reactive oxygen species (ROS), previously considered intracellular byproducts of aerobic metabolism, are now known to regulate multiple signaling pathways as second messengers. Cancer cells cope with elevated amounts of ROS during therapy by upregulating the antioxidant system, enabling tumor therapeutic resistance *via* a variety of mechanisms. In this review, we aim to shed light on redox modification and signaling pathways that may contribute to therapeutic resistance. We summarized the molecular mechanisms by which redox signaling-regulated drug resistance, including altered drug efflux, action targets and metabolism, enhanced DNA damage repair, maintained stemness, and reshaped tumor microenvironment. A comprehensive understanding of these interrelationships should improve treatment efficacy from a fundamental and clinical research point of view.

## 1 Introduction

Although the risk of death from cancer has decreased continuously by 32% over the past 3 decades, cancer treatment remains a significant global public health issue that faces multiple challenges (Siegel et al., 2022). In the early stage of tumor development, most patients will respond completely or partially to chemotherapy, whereas in the late stage, the therapeutic effect is still unsatisfactory, mainly due to drug resistance (Miller et al., 2019). Drug resistance is generally divided into inherent resistance and acquired resistance. Specifically, intrinsic resistance is mainly caused by existing factors (e.g., gene mutations), while acquired resistance is caused by changes in tumor cells during treatment, including drug target expression, post-translational modification, and activation of bypass signaling (Holohan et al., 2013; Li et al., 2020). Recent studies have shown that the stress response induced during tumor treatment, especially oxidative stress, is one of the vital reasons for the acquired drug resistance of tumor cells (Cui et al., 2018; Qin et al., 2022).

Traditional and new treatments, including surgery, immunotherapy, radiotherapy, chemotherapy, and targeted therapy, are the main methods of cancer treatment at present (Ward et al., 2021). Among several novel therapeutic approaches to overcome drug resistance, manipulating redox homeostasis is efficient in various tumors (Cui et al., 2018; Hayes et al., 2020). The regulation of redox homeostasis is essential in maintaining cell survival and normal function, which is accomplished through a balance of Reactive oxygen species (ROS) production and elimination (Lennicke and Cochemé, 2021). ROS in cells are mainly derived from the mitochondrial electron transport chain, endoplasmic reticulum oxidase, and NADPH oxidase (NOXs) (Block and Gorin, 2012; Ashton et al., 2018; Jakubczyk et al., 2020). The elimination of ROS is accomplished by antioxidants, antioxidant enzymes, antioxidant transcription factors NRF2, and high abundance redox proteins (Harris and DeNicola, 2020). NRF2 is a transcription factor involved in cell homeostasis and plays a crucial role in regulating redox homeostasis, drug metabolism, energy metabolism, proteasome degradation, and other physiological processes (Huang et al., 2015). Meanwhile, NRF2 targets genes associated with cell defense, including antioxidant response elements (AREs) (Lu et al., 2016). However, in oxidative stress cells, oxidation of the KEAP1-Cul3-RbX1 complex destroys specific key cysteine residues in Keap1, thereby interfering with the ubiquitin degradation process of NRF2, resulting in enhanced transcriptional activation of genes, including GSH, heme oxygenase-1 (HO-1), NADP(H), and quinone oxidoreductase 1 (NQO1) (Itoh et al., 1997), and the enhancement of chemotherapy resistance (Wang et al., 2008). ROS can act as a double-edged sword: In normal cells, the activation of ROS signaling can reduce the possibility of the formation of cancerous lesions; but its abnormal activation in precancerous cells with high levels of ROS may benefit cancer cell survival (Hayes and McMahon, 2006; Lau et al., 2008; Wang et al., 2008; Sporn and Liby, 2012; Gañán-Gómez et al., 2013). When the production and elimination of intracellular ROS are in a state of dynamic equilibrium, the cells may survive and become redox homeostatic. Previously considered intracellular by products of aerobic metabolism, reactive oxygen species (ROS) have now been seen as second messengers regulating multiple signaling pathways (Forman, 2016). The disulfide bond, a chemical bond formed by sulfhydryl groups connecting two different cysteine residues in the peptide chain, can stabilize the spatial configuration of various receptors, enzymes, and other proteins (Hohl et al., 2012). It has been found that redox homeostasis of cells has a vital influence on the formation of reversible disulfide bonds and plays a crucial role in spatial structure and functional maintenance (Liu et al., 2016). Changes in redox modifications and redox-mediated pathways together mediate oxidative stress-induced tumor drug resistance.

Under normal physiological conditions, the body is in a state of redox homeostasis, that is, the balance between oxidants and antioxidants. Compared to normal cells, cancer cells react with higher ROS levels during treatment through upregulating their antioxidant system. This promotes tumor therapeutic resistance *via* various mechanisms, such as altered drug efflux, metabolism and targets, improved DNA damage repair, preserved stemness, and modified tumor microenvironment. A comprehensive understanding of their relationships should improve treatment efficacy from a fundamental and clinical research point of view. This review aims to summarize the molecular mechanisms of redox-regulated cancer drug resistance and explore potential clinical strategies to overcome drug resistance.

## 2 Redox signaling regulates drug efflux

Sufficient intracellular drug concentration is required for drug toxicity that can effectively kill tumor cells, and plasma membrane transporters regulate drug concentration by regulating drug influx and efflux. Drug influx is characterized by drug concentration on both sides of the cell membrane, the aid of drug transporters, and the requirement of energy (Joyce et al., 2015). Drug efflux has been widely studied in numerous species as a crucial intracellular detoxification mechanism (Mandal et al., 2017).

Several cell membrane transporters are crucial to chemotherapeutic efflux and influx and drug resistance. As defined by the main amino acid sequence, they all have widespread substrate molecular specificity, promoting the elimination of major cancer chemotherapeutic agents such as antimetabolites, taxanes, and topoisomerase inhibitors (Wong et al., 2014). Multidrug resistance protein 1 (MDR1), MDR-associated protein 1 (MRP1), and breast cancer resistance protein (BCRP) are three of them that have been widely examined in relation to cancer drug resistance to chemotherapy (Sharom, 2008; Fletcher et al., 2010). ATP-binding cassette (ABC) transporters serve on efflux pumps that actively transport drugs out of cells against concentration gradients, thus promoting MDR by increasing the efflux of anticancer drugs so that the intracellular drug concentration is less than the level of effective drug therapy (Yardley, 2013; Li et al., 2016). ROS modulate ABC transporters in a two-pronged way: moderate oxidative stress may be necessary for the induction of ABC transporters in tumor cells, whereas excessive oxidative stress may inhibit the transporter system (Yuan et al., 2022).

### 2.1 Redox changes the conformation of drug efflux transporters

Specifically, ABC transporters contain two cytoplasmic nucleotide-binding domains (NBDs) and two transmembrane domains (TMDs), which can be merged into multidomain peptides in a great diversity of ways. Conversion between the two main conformations of NBD dimers: ATP binding induces each NBD to form a closed dimer, and ATP hydrolysis restores the dimer to an open configuration. Changes in the two configurations generate driving forces that facilitate drug transport, reducing intracellular drug levels to sublethal (Higgins, 2007).

The transport activity of MDR1 is related to the REDOX state of cysteine residues Cys431 and Cys1074 in NBD1 and NBD2, and the structures of the two binding sites are similar. MRP1 also has a topological structure, in addition to a transmembrane domain MSD0, which acts as a gate valve in the process of drug transport across the membrane. Cysteine residues in MSD0 establish disulfide bonds to dimerize MRP1, and their structural mutations may prevent dimer formation and interfere with drug transport. For instance, cysteine residues in MSD0 can be affected by drug induction (e.g., destruction of MRP1 by the reducing agent dithiothreitol (DTT)) (Maiti, 2012). Furthermore, ROS can be produced after treatment with some anticancer drugs to promote the upregulation of the antioxidant system in cancer cells, such as enhancing the expression of GSH. The cysteine residue of glutathione is its active group, which can bind to a multitude of drugs and toxins and promote the efflux of drugs through MRP1, thus having an integrated detoxification effect (Krause et al., 2007).

Human BCRP, belonging to ABCG2, is a semi-molecular transporter responsible for the delivery of multiple drugs, including mitoxantrone and SN-38. BCRP plays a key role in chemotherapeutic resistance as a homodimer drug transporter. Notably, there are three important amino acid residues in BCRP, Cys592, Cys603, and Cys608, located on the outer surface of the plasma membrane (Henriksen et al., 2005). ABCG2 cysteine residues Cys592 and Cys608 formed intramolecular disulfide bonds, regulating substrate specificity, whereas Cys603 and Cys608 created intermolecular disulfide bonds, determining plasma membrane localization (Wakabayashi et al., 2006). Intermolecular and intramolecular disulfide bridges are critical to the structural and functional integrity of ABCG2. According to research, when cysteine residues are muted owing to external factors, the spatial structure of the BCRP protein changes, affecting its activity and function to overcome multidrug resistance and improve drug sensitivity (Kage et al., 2005). ROS regulation of ABC transporters is a new method to overcome drug resistance in cells. These studies suggest that ROS inactivate transporters by affecting disulfide bonds in the transporter protein dimer, highlighting the potential of ROS to alter transporters to reduce drug efflux and thereby promote drug efficacy.

### 2.2 Redox altered transporter gene expression

Studies have shown that redox regulation of drug efflux transporters can be carried out through multiple pathways, and several transcriptional pathways that regulate gene expression induced by environmental factors have been identified (McMahon et al., 2003). ROS mediated by endoplasmic reticulum stress have been shown to upregulate MDR1 expression in hepatoma cells (Ledoux et al., 2003). In Caco-2 human colon cancer cells, MDR1 expression is increased under high-concentration treatment with H_2_O_2_ (Terada et al., 2014). In detail, the development of oxidative stress leads to the oxidation of the SH group, which upregulates NRF2. NRF2 mediates an increase in the content and activity of p-GP transporters, thereby limiting the increase in membrane permeability (Shchulkin et al., 2021). In response to NRF2 activation, fork-head box O (FOXO) can be activated through disulfide interactions with transporters. According to the study by Myatt and Lam. (2007), the inactivation of the transcription factor FOXO may lead to cell carcinogenesis, so the activation of FOXO may contribute to the expression of drug efflux transporters (Gomes et al., 2013). After chemotherapeutic agents are used, intracellular redox equilibrium is disrupted and ROS accumulate, promoting the activation of FOXOs and inducing the expression of transporter proteins. For example, paclitaxel (PTX) has been reported to induce drug resistance in ovarian malignant tumor cells *via* Trx1 and the transcription factor FOXO (Wang et al., 2015). Furthermore, oxidative stress can promote the transcription of multiple transporter protein genes, including MDR, MRPs, and BCRP, by promoting the translocation of apurinic-apyrimidinic endonuclease I (APE-1) or redox factor I (Ref-1). In addition, oxidative stress network activation affects disulfide bonds between PKA or PKC to increase the activity of protease, which in turn affects its oxidative phosphorylation substrate MDR1, thereby reducing cellular drug sensitivity (Blobe et al., 1993; Brennan et al., 2006; Kang and Yang, 2020). Moreover, there is increasing evidence that epigenetic regulation can influence drug resistance by altering the configuration of drug efflux pumps. For example, methylation of the MDR1 promoter region silences the transcription of its genes (Lertratanangkoon et al., 1997; Baker et al., 2005). Overall, oxidative stress affects drug efflux *via* redox modification and redox signaling; hence, an in-depth understanding of the molecular mechanism can aid in resolving the clinical drug resistance issue.

## 3 Redox homeostasis in tumor cell survival and death

### 3.1 DNA repair

DNA can be damaged by various endogenous and environmental agents, leading to its physical or chemical changes (Chatterjee and Walker, 2017). Although oxidative stress can damage cellular structures, DNA damage and genomic instability in the initial stage can drive the accumulation of carcinogenic changes that contribute to the development of cancer (Basu and Nohmi, 2018). Studies have found that ROS, as DNA damage agents during tumorigenesis, effectively increases cell mutation rate, thereby promoting carcinogenic transformation (Jackson and Loeb, 2001). ROS can generate DNA lesions in many ways, including base modification, nucleotide removal, DNA structural disruption, and DNA-protein crosslinking (Barnes et al., 2019). For example, ROS induce DNA fragmentation in sperm nuclear/mitochondrial genomes and reduce transcription levels (Bisht and Dada, 2017). Guanine is more easily oxidized than any other DNA base, and 8-hydroxy-2′-deoxyguanosine is the most usual form of oxidized guanine (Salehi et al., 2018). In addition, ROS can indirectly cause extra cyclic DNA damage by inducing lipid peroxidation (Yu et al., 2016). Oxidative stress also leads to mitochondrial DNA base lesions and degradation, an essential factor in mitochondrial gene mutations (Shokolenko et al., 2009).

The cell response to DNA damage is a complex process involving a variety of signaling networks and proteins that are activated or inactivated differentially in specific cancer types (Salehi et al., 2018). For a series of DNA damage events, from single-strand breaks (SSBs) to base alkylation events, tumor cells will initiate the corresponding DNA damage response (DDR) to resist this effect, thus maintaining tumor survival (Curtin, 2012; Lord and Ashworth, 2012). In normal cells, spontaneous DNA damage and repair are roughly balanced, and most DNA damage is repaired correctly. In tumor cells, however, this balance may change due to inadequate repair ability (Srinivas et al., 2019). Experiments have shown that ROS inhibit the activation of the DNA repair enzyme OGG1 (an 8-oxoG DNA glycosylase) by oxidizing critical cysteine residues such as Cys253 and Cys255 (Bravard et al., 2006). In addition, ROS can delay the recognition of damaged areas by affecting a range of sensor kinases (ATM and ATR) and downstream sensor kinases (CHK1 and CHK2) (Chang et al., 2015). H2AX has been shown to cluster around damage sites under the regulation of CTCF in the case of DNA damage, thus initiating DNA repair. Continuous oxidative stress accelerates the degradation of H2AX interceded by the E3 ubiquitin ligase RNF168, which enhances DNA damage in cancer cells (Gruosso et al., 2016). As ROS can inhibit the DDR of tumors to a certain extent, treatment with increased ROS may promote the death of cancer cells.

### 3.2 Apoptosis

Apoptosis is an evolutionarily conserved process that is essential for development and homeostasis. Furthermore, apoptosis is regulated by two main pathways, the death receptor pathway and the mitochondrial pathway, with ROS strongly implicated in the signal transduction of these pathways (Circu and Aw, 2010). Small amounts of ROS facilitate cancer cell development by activating several key factors in the cell cycle. A large amount of ROS generate oxidative stress that triggers programmed cell death, or apoptosis (Johar et al., 2015). In the case of tumors, cells lose their ability to undergo normal apoptotic induction, leading to uncontrolled proliferation (Mohammad et al., 2015). Studies have shown that VB1 increases the level of ROS in anti-BRAFi melanoma cells, leading to DNA cytotoxicity, G2/M cell cycle stagnate, and apoptosis (Liu et al., 2018). Bcl-2 family proteins are key factors regulating apoptosis, and their expression and transport to mitochondria are regulated by ROS (Li et al., 2004). After treating HepG2 cells with zinc oxide nanoparticles, reactive oxygen species changed mitochondrial membrane potential, degraded and downregulated Bcl-2, resulting in cell apoptosis (Sharma et al., 2012). In addition, doxorubicin promotes ROS accumulation in keratinocytes, inactivating ERK1/2 by oxidative modification of cysteine sulfonic acid, leading to the downregulation of Bcl-2 and induction of apoptosis (Luanpitpong et al., 2012). After sodium nitroprusside treatment, S-nitrosylation modification at Cys183 decreases the phosphorylation of ERK1/2, thus promoting apoptosis (Jin et al., 2018).

In various malignancies, accumulated ROS may activate p53 through the JNK signaling pathway, thereby promoting apoptosis (Shi et al., 2014). The cytosolic protein c-FLIP (cellular FLICE-inhibitory protein) is a death receptor-mediated apoptosis inhibitor that is upregulated in various cancers, thereby promoting the anti-apoptotic ability of tumor cells. ROS can affect the structural stability of the protein and promote its degradation (Wilkie-Grantham et al., 2013; Ming et al., 2023). In conclusion, increasing the ROS content in tumors to a certain extent through drugs may promote tumor cell apoptosis, thus reducing tumor drug resistance.

### 3.3 Necroptosis

Programmed cell necrosis is a mode of cell death mediated by death receptors and is closely regulated by intracellular signaling factors. This process involves the self-destruction of cells activated to prevent cell apoptosis from being blocked (Dhuriya and Sharma, 2018). At the core of necroptosis signaling is the activation of protein kinase receptor-interacting protein 3 (RIP3) by an upstream protein with the RIP homotypic interaction motif (RHIM) domain. This phosphorylates and activates mixed lineage kinase domain-like (MLKL), leading to cell membrane destruction and necrosis (Dondelinger et al., 2016). Ligands such as TNF and FasL can induce necroptosis, causing cell swelling and rupture. This releases damage-related molecular patterns (DAMPs), which are detected by the innate immune system as danger signals and trigger inflammatory responses (Orozco and Oberst, 2017).

Necroptosis has been associated with the inhibition of tumor development in some cancer types. For example, dysregulation of necroptosis has been linked to the development of acute myeloid leukemia and ovarian cancer. However, for esophageal, pancreatic, and colon cancer, necroptosis promotes tumor progression and metastasis. The underlying mechanism remains elusive (Wang et al., 2017). Studies have suggested that necroptosis is closely related to ROS and that they are positively correlated, which plays an important role in pathophysiological conditions such as tumors (Hsu et al., 2020). One study showed that ROS-mediated modification of residues of cysteine 257, 268, and 586 can lead to the formation of an intramolecular disulfide bond on RIP1, which promotes the autophosphorylation of RIP1 at serine 161 thereby effectively recruiting RIP3. These results suggests that ROS serves as an inducer of necroptosis (Zhang Y. et al., 2017). It was found that under hydrogen peroxide treatment, RIP3 could migrate to the endoplasmic reticulum and induce calcium ion imbalance, suggesting that oxidative stress could initiate programmed necrosis of cardiomyocytes through upregulation of RIP3 (Zhang T. et al., 2016). In addition, removal of ROS with butylated hydroxyanisole significantly reduced TNF-mediated necrosis in a mouse fibrosarcoma L929 cell line (Vanlangenakker et al., 2011). Although the molecular mechanism between necrosis and ROS has been established, effective therapies to inhibit cancer progression by simultaneously regulating necrosis and ROS need further investigation.

### 3.4 Autophagy

Autophagy is an intracellular catabolic process. Under nutrient deficiency or stress stimulation, the cell degrades misfolded proteins and damaged organelles, thereby providing the cell with energy (Klionsky et al., 2021). For tumors, the role of autophagy is two-sided and complex. In general, autophagy mainly inhibits tumors in the early stage of tumor formation, while it specifically protects and helps tumor cells survive (Rebecca and Amaravadi, 2016). Studies have shown that REDOX signal transduction and modification are closely related to autophagy. For example, ROS can regulate mTOR activity through various pathways, such as the LKB1/AMPK, HMGB1, and PI3K/Akt pathways, thus inducing autophagy (Gibson, 2013). ROS can also activate autophagy by phosphorylating JNK in human breast cancer cells (Sun et al., 2017). In addition, numerous autophagy-related proteins, including LC3-II, P62, and ATG protein, were activated in H9c2 cells treated with H2O2 (Ha et al., 2012). In laser therapy of cancer, accumulated ROS can increase beclin-1 expression, thereby promoting tumor autophagy and increasing its therapeutic resistance (Shu et al., 2016). However, several studies have shown that oxidative modifications of autophagy regulatory factors play an inhibitory role in autophagy. Oxidation of the autophagic proteins ATG4B on cysteine Cys292 and Cys361 inhibited the activity of tubulin 1A/1B LC3, leading to reduced autophagic flux (Zheng et al., 2020). In addition, oxidative modification of Cys263 and Cys572 inhibits LC3 lipidation (Frudd et al., 2018).

In addition to the two aspects of the influence of oxidative stress on autophagy, autophagy may mediate a negative feedback loop to prevent oxidative stress. Elevated ROS induces Ca^2+^ release by activating MCOLN1, leading to nuclear translocation of TFEB, which induces autophagy. Enhanced autophagy promotes the elimination of excess ROS (Zhang X. et al., 2016). If autophagy is insufficient to remove excess ROS, it may cause the death of tumor cells. Providing new targets may help accelerate the process of drug-resistant tumor therapy, and the specific role of oxidative stress on autophagy needs to be verified by further studies.

### 3.5 Cell survival

**FIGURE 1:**
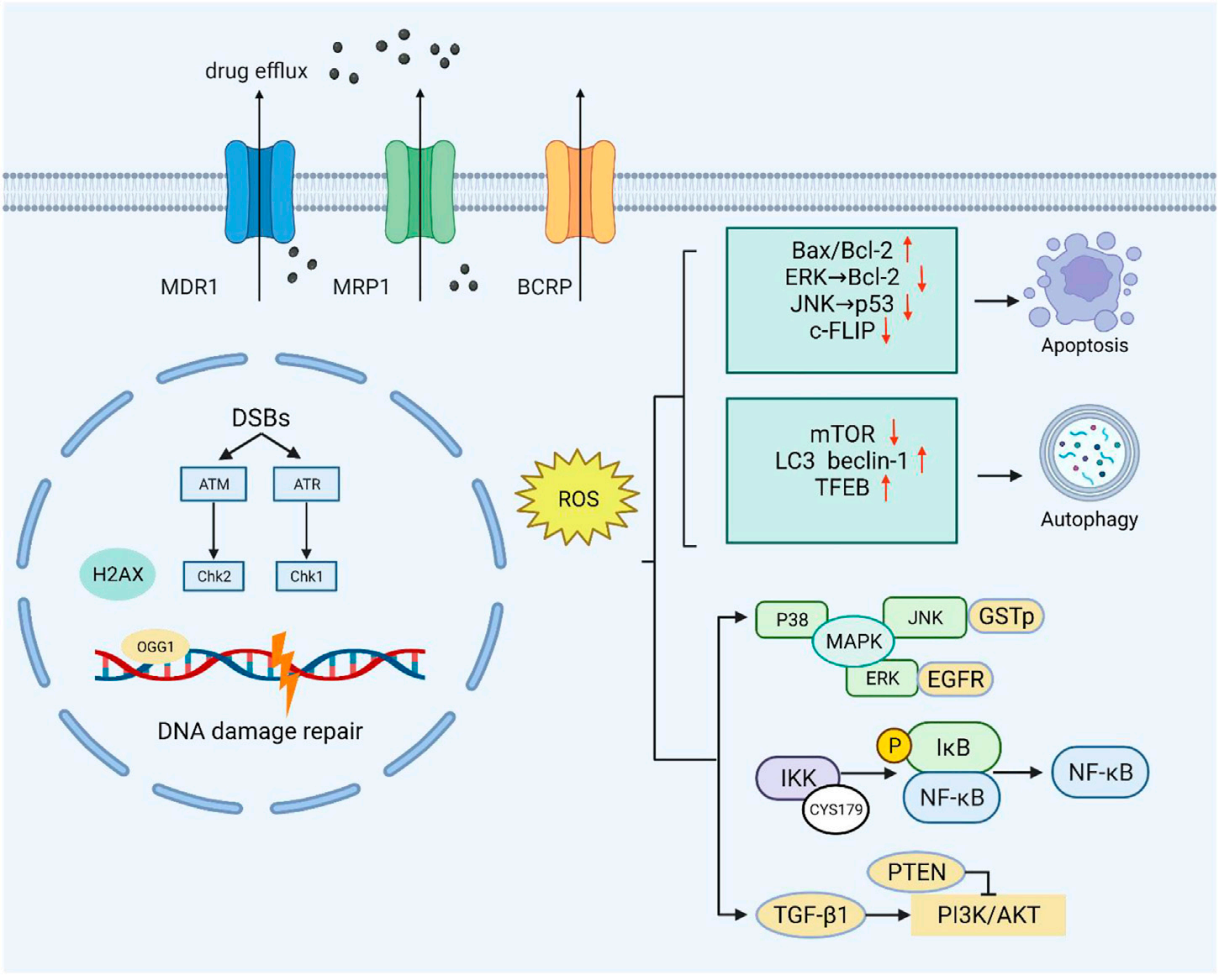
Moderate ROS levels can limit the anticancer activity of drugs by increasing drug efflux. Excessive ROS can cause endogenous DNA damage and inhibit the DNA damage repair response. At high ROS levels, death receptor-mediated and mitochondrial pathways are triggered to induce apoptosis. ROS can also inhibit the negative regulators of autophagy (mTOR) and increase the formation of autophagosomes by upregulating autophagy-related genes. At low levels, ROS act as second messengers by mediating multiple signaling pathways (PI3K/AKT, MAPK, and NF-κB) that contribute to cancer cell survival and proliferation.

Moderate ROS promote cell survival and proliferation by means of multiple pathways, such as NF-κB, MAPK, and PI3K/AKT, which play a key role in tumor drug resistance (Figure 1). Activation of PI3K can lead to cell growth, cell proliferation, cell survival, etc. Modification of this pathway is closely related to the pathogenesis of most cancers (Noorolyai et al., 2019). The tumor suppressor gene PTEN is a negative regulator of the PI3K/AKT signaling pathway. Studies have found that oxidative stress can induce the inactivation of PTEN (oxidation on Cys124 and Cys71), thereby activating the PI3K and AKT pathways to promote cell survival (Leslie et al., 2003; Kim et al., 2018).

The ROS-dependent activation of TGF-β1 was specific and restricted to the oxidation of Met253 on latency-associated peptide β (LAP-β) (Richter et al., 2015). Studies have shown that ROS upregulates and activates the PI3K/Akt signaling pathway through TGF-β1, leading to malignant changes in kidney cells (Lu et al., 2019). Intriguingly, PI3K/Akt also upregulates glutathione synthesis through NRF2 to resist oxidative stress in breast tumors (Lien et al., 2016).

NF-κB plays an irreplaceable role in cell survival and proliferation, and NF-κB activation can actuate drug resistance in a variety of tumors during treatment (Drain et al., 2021). Oxidative stress has the potential to regulate NF-κB activity, but the mechanism is not simple. Studies have shown that ROS can both promote and inhibit NF-κB activity, and in most cases, NF-κB activity is elevated in tumor cells (Nakajima and Kitamura, 2013). Berbamine-mediated excess ROS negatively regulates the NF-κB pathway by means of activation of NF-κB inhibitor (IκB) and oxidation of cysteine in the binding region of NF-κB to DNA, thereby inhibiting the development of bladder cancer (Han et al., 2021). In addition, ROS-mediated oxidation of Cys179 can inhibit IKK activity, which is essential for NF-κB activation (Reynaert et al., 2006).

The mitogen-activated protein kinase (MAPK) signaling pathway has four subfamilies: ERK, P38, JNK, and ERK5, which mediate reactions from plentiful stimuli (Latimer and Veal, 2016). Oxidative modification of Cys119 and Cys162 of p38 affected the dimerization of its key upstream activator mitogen-activated protein kinase 3 (MKK3) (Bassi et al., 2017). ROS have been shown to activate EGF receptors, which can stimulate the subsequent activation of the ERK signaling pathway (León-Buitimea et al., 2012). Recently, studies have shown that ginsenoside Rh4 can activate autophagy and apoptosis in colorectal tumor cells by activating the ROS/JNK/p53 pathway (Wu Q.et al., 2018). In addition, ROS can separate JNK from glutathione S-transferase pi (GSTp), which can inhibit the activation of JNK, thereby facilitating the JNK signaling pathway (Castro-Caldas et al., 2012).

In summary, the redox-mediated cell survival signaling pathway significantly supplements drug resistance mechanisms. Further research on ROS targets will help speed up the development process of studying the molecular mechanism of ROS-induced tumor resistance and formulate therapeutic strategies.

## 4 Stress homeostasis and drug resistance

### 4.1 Redox homeostasis

Tumor cells can enter a reversible drug-tolerant persister (DTP) state to avoid chemotherapy and targeted drug attack by a mechanism different from tumor resistance through genetic mutations. In the case of oxidative stress caused by drug stimulation, DTP cells rely heavily on regulating redox homeostasis to avoid cell death, which is regarded as a major cause of tumor drug resistance (Mikubo et al., 2021). On the one hand, tumor cells with acquired drug resistance can increase oxidative stress and maintain their active proliferation state. On the other hand, tumor cells can also activate the antioxidant defense system to avoid oxidative stress-mediated cell death (Chini et al., 2021). It was found that oxidative stress can promote NRF2-mediated activation of the antioxidant genes. Keap1 also inhibits the phosphorylation of IκBα by mediating IKKβ degradation, thus antagonizing oxidative stress induced by the NF-κB pathway. Oxidative stress also promotes FOXO entry into the nucleus and activates the expression of downstream antioxidant genes (Rojo de la Vega et al., 2018; Hayes et al., 2020). Glutathione (GSH), a crucial reductive force in cells, can significantly reduce oxidative damage and lead to tumor drug resistance (Bansal and Simon, 2018). In detail, cystine transporter xCT can promote the transport of cysteine into the cell and provide the raw material for GSH synthesis. Recent research suggested that oxidative stress can enhance the expression and activity of xCT through transcription and post-translational modification, thus affecting the synthesis of GSH (Daher et al., 2019). In addition, the CD44 variant promotes intracellular cystine transport and GSH synthesis by interacting with cystine transporter xCT, thus maintaining very low ROS levels in tumor stem cells (Ishimoto et al., 2011). In addition, some high-abundance redox proteins can buffer oxidative stress through non-classical antioxidant pathways mediated by self-oxidation modifications. For example, the active cysteine sites (Cys115 and Cys161) of CypA undergo oxidative modification to form intramolecular disulfide bonds, which contribute to the elimination of intracellular ROS. Further studies have found that PRDX2 can bind to CypA through disulfide bonds and promote the reduction of oxidized CypA, thus maintaining the reduction ability of colorectal cancer cells (Peng et al., 2021). Drug-resistant cells express the sterol lipid transporter NPC1L1 (Niemann-Pick C1-like 1) to compensate for the inhibition of GSH synthesis caused by the downregulation of xCT. Meanwhile, NPC1L1 can resist oxidative stress induced by tumor therapy to a certain extent by promoting the uptake of vitamin E and cholesterol (Zhang et al., 2022). ANXA2 can also enhance tumor cell survival by buffering excessive oxidative stress through oxidative modification and inducing autophagy (Wang et al., 2018). The above studies indicate that DTP cells can resist oxidative stress induced by tumor therapy by regulating intracellular redox-related pathways, which provides ideas for targeting antioxidant systems to overcome tumor drug resistance.

### 4.2 Metabolic reprogramming

**FIGURE 2:**
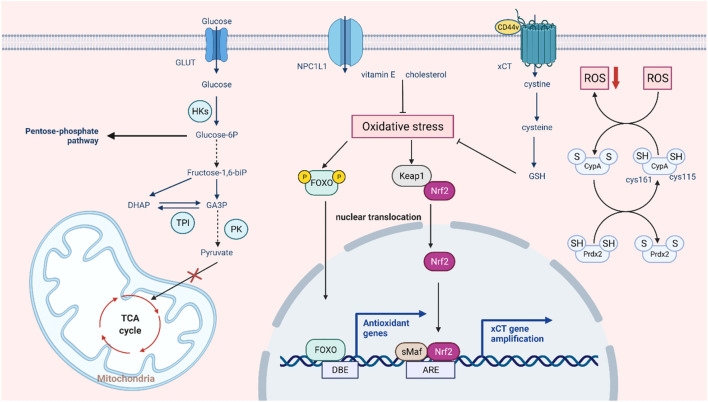
Intrinsic pathways involved in ROS-mediated drug resistance. In the aerobic state, tumor cells still preferentially choose glycolysis and conversion to lactic acid, and ROS regulate the three key enzymes in this process. In response to excessive oxidative stress, the glycolytic pathway partly switches to the pentose phosphate pathway, and the tumor also activates the antioxidant system to prevent cell death by allowing the translocation and activation of NRF2 and FOXO and enhancing XCT transport function to promote GSH synthesis. In addition, some non-classical antioxidant pathways, such as autooxidation modification of CypA and compensatory transport of NPC1L1, can also buffer oxidative stress.

In somatic cells, glucose is metabolized by the tricarboxylic acid cycle and then phosphorylated to provide energy. When the oxygen supply is insufficient, a large amount of pyruvate is produced by anaerobic glycolysis and converted to lactic acid to maintain the energy supply. Even when oxygen is abundant, tumor cells preferentially conduct glycolysis to meet the demand for energy and nutrients for rapid proliferation, a phenomenon known as the Warburg effect (Liberti and Locasale, 2016). This metabolic change is widely regarded as a hallmark of cancer (Potter et al., 2016). A growing body of research suggests a link between aerobic glycolysis and tumor growth and chemotherapy resistance (Sattler et al., 2010). In addition, this effect may not be due to defective mitochondrial respiration but to the upregulation of glycolytic enzymes and glucose transporters (Wangpaichitr et al., 2021). While intracellular ROS production and the maintenance of redox homeostasis largely depend on cellular metabolism, ROS can also affect tumor energy metabolism by regulating key metabolic enzymes (Quijano et al., 2016). Many glycolytic enzymes contain highly conserved cysteines that are susceptible to redox modification in response to ROS (Lennicke and Cochemé, 2021). Hexokinase (HK) is the first rate-limiting step in catalyzing glucose metabolism, promoting the immediate use of ATP (Heneberg, 2019). Using 2-DG as an HK2 inhibitor to sensitize tumor cells can improve the efficacy of the cytotoxic drugs doxorubicin and paclitaxel (Maschek et al., 2004). It has been found that dehydroascorbic acid (DHA) can covalently bind to the active cysteine of hexokinase 1 (HK1) and irreversibly lose its enzyme activity (Fiorani et al., 2000). Pyruvate kinase M2 (PKM2) is a glycolytic enzyme with less pyruvate kinase activity that can prevent the flow of glycolytic metabolites into the TCA cycle, thus maintaining the survival of tumor cells (Christofk et al., 2008). Silencing PKM2 can increase docetaxel accumulation in A549 lung cancer cells and enhance its antitumor effect (Shi et al., 2010). In lung cancer cells, hydrogen peroxide caused a stepwise increase in ROS levels and further inhibited PKM2 activity by oxidizing cysteine Cys358 (Anastasiou et al., 2011). Dihydroxyacetone phosphate (DHAP) isomerizes under the catalytic action of triose phosphate isomerase (TPI) to form glyceraldehyde-3-phosphate (G3P) (Pekel and Ari, 2020). It has been found that ROS may cause oxidation of TPI by affecting intramolecular disulfide bonds, and oxidative TPI is subsequently degraded, thus turning glycolysis into the pentose phosphate pathway (PPP) (Dumont et al., 2016). Elevated levels of GAPDH are considered a key factor in the maintenance of tumor glycolytic phenotypes. Under oxidative stress, Cys152 of GAPDH can be oxidized to form intermolecular disulfide bonds, leading to aggregation of GAPDH and cell death (Reisz et al., 2016). In addition, redox modification of cysteine inhibits the activity of GAPDH and shifts metabolic flux from glycolysis to the PPP pathway (Ralser et al., 2007). Since cancer cells generally express more ROS than normal cells, changes in glycolysis to the pentose-phosphate pathway enhance the reductive ability of tumor cells to some extent (Kuehne et al., 2015). In this case, ROS act as signaling molecules that regulate tumor metabolism in response to changes in the cellular environment. This also suggests that the combination of ROS regulators and metabolic recombination of tumor cells can provide a new idea for tumor therapy. In conclusion, ROS-mediated redox modification can regulate the function of metabolic enzymes, thereby regulating tumor metabolic reprogramming and playing a significant role in tumorigenesis and development (Figure 2).

## 5 EMT and cell stemness regulated by redox signaling

**FIGURE 3:**
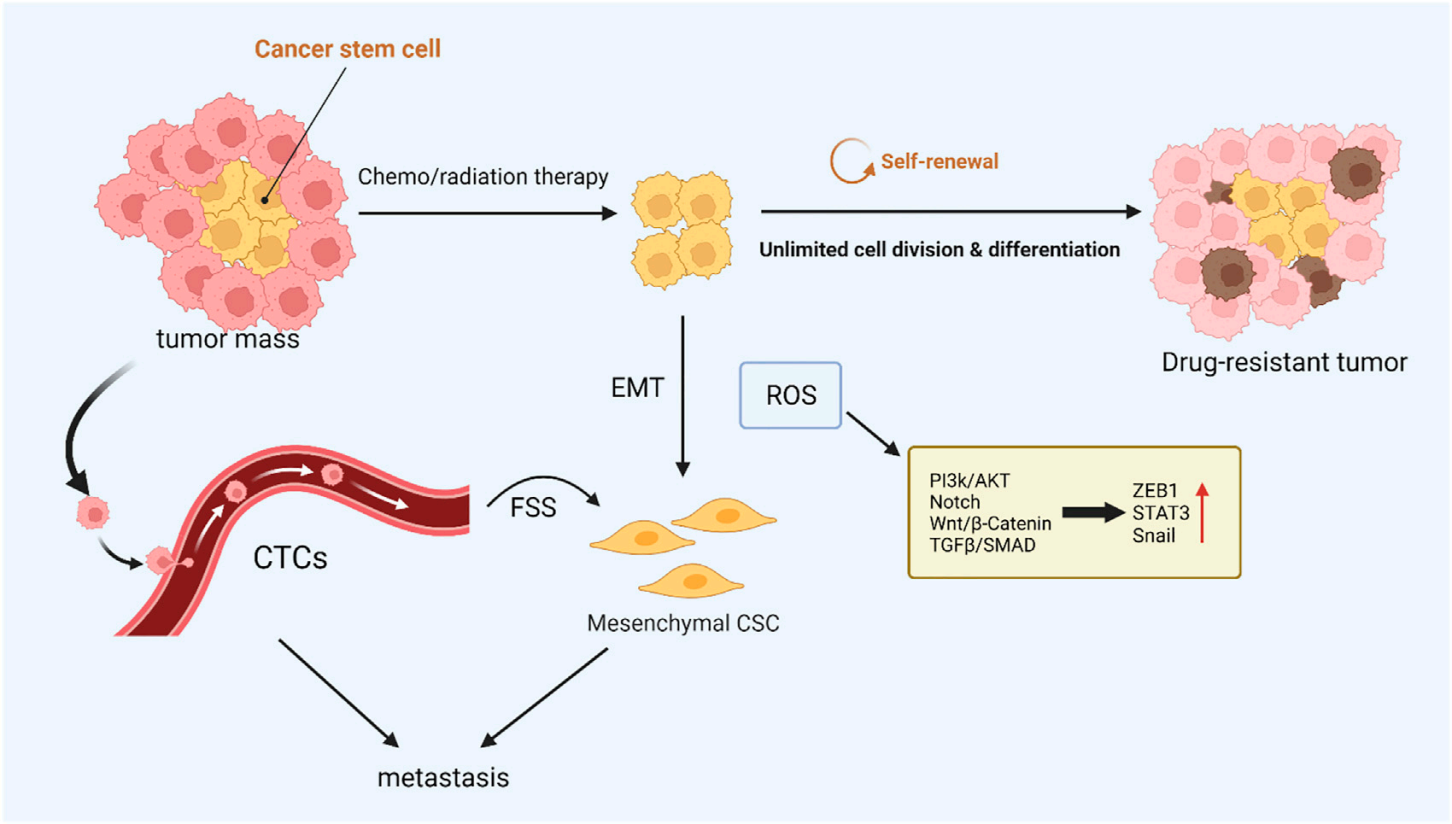
ROS-mediated cancer cell stemness. CSCs with low intracellular ROS levels are resistant to chemo- or radiotherapy, and CTCs are closely related to tumor metastasis. These resistant CSCs may contribute to local recurrence or metastasis *via* EMT. ROS can drive both EMT and stem cell phenotypes, and the activated signaling pathways are similar.

Epithelial-to-mesenchymal transition (EMT) plays a pivotal part in inducing tumor metastasis and promoting drug resistance (Chang, 2016; Ming et al., 2021). During EMT, epithelial cells lose their intercellular adhesion and cell polarity characteristics, as well as acquire motility, migration potential, and invasiveness (Brabletz et al., 2018). Cancer stem cells (CSCs) take an essential effect on tumor survival, proliferation, and metastasis. Specifically, CSCs can maintain cancer cell activity utilizing continuous self-updating and limitless proliferating, which are closely related to tumor drug resistance and recurrence (Phi et al., 2018). It was found that the signaling pathways activated in EMT are significantly similar to those that drive CSCs, such as the Wnt, Hedgehog, and Notch signaling pathways, which suggests that EMT is closely related to stem cell status in normal and tumor epithelial tissues (Du and Shim, 2016). In ionizing radiation, ROS activate a variety of EMT transcription factors, including ZEB1, Snail, STAT3, and HIF-1, thus promoting the metastasis and invasion of tumor cells (Lee et al., 2017). In breast cancer cells, poly ADP-ribose polymerase 3 (PARP3) plays an important role in inducing EMT and CSC phenotypes by regulating the TG2-snail-E-cadherin axis in response to ROS (Karicheva et al., 2016). It has been found that nicotine-induced elevated ROS levels alter EMT by regulating the AKT pathway in human renal epithelial cells to a certain extent and form stem cell-like spheroids (Chang and Singh, 2019). Paradoxically, after tumor stem cells are formed, intracellular R OS levels are low, which helps maintain the characteristics of CSCs (Qian et al., 2018). In addition, the stemness of CSCs was successfully reduced by increasing intracellular reactive oxygen species, and the efficiency of cisplatin therapy was improved (Chang et al., 2014). EMT-affected cells move from the primary tumor site to the surrounding stromal environment. The next is endovascular osmosis, which involves cancer cells entering the circulation and then being transported to distant organs. This process is closely linked to circulating tumor cells (CTCs) (Shibue and Weinberg, 2017). CTCs refer to tumor cells that are shed from solid tumor lesions and enter the bloodstream during tumor formation and development, reflecting tumor development and metastasis (Paoletti and Hayes, 2016). In blood circulation, the environmental factor that CTCs need to deal with is fluid shear stress (FSS), which can cause ROS generation, thus motivating CTCs to increase proliferation and behave more stem-like (Brown et al., 2021). The ERK pathway activates FSS-induced breast cancer cell survival, migration, and stemness alteration (Ma et al., 2017). In addition, ROS generated by PMN-MDSCs can promote the survival of CTCs by inducing the activation of the Notch signaling pathway (Sprouse et al., 2019). Different levels of ROS have different effects on the generation and maintenance of tumor stemness and the process of metastasis, which requires further studies to regulate ROS concentration to overcome drug-resistant tumors accurately (Figure 3).

## 6 Tumor microenvironment and redox homeostasis

The tumor microenvironment comprises tumor cells, stromal cells, immune cells, secretory products (such as cytokines and chemokines) of corresponding cells, and non-cellular components in the extracellular matrix (ECM), which are closely related to tumor growth and metastasis. Hypoxia is one of the most prominent features of the tumor microenvironment and is associated with many tumor characteristics, including promoting cell proliferation, angiogenesis, metabolic reprogramming, and resistance to radiotherapy and chemotherapy (Jing et al., 2019; Bao and Wong, 2021). Reduced oxygen availability reduces the flow of electrons through the mitochondrial complex, allowing electrons to leak out of the transport chain and leading to the overproduction of ROS (Guzy et al., 2005). Angiogenesis in the tumor microenvironment is essential for cancer cell expansion and metastasis. Some evidence suggests that reactive oxygen species enhance angiogenesis. Under hypoxia, tumor cells express various angiogenic factors, such as vascular endothelial growth factor (VEGF), by activating HIF proteins (Xia et al., 2007). It has been found that inhibiting the expression of angiogenic markers and other oncogenic factors by reducing ROS can effectively suppress angiogenesis and dramatically inhibit tumor growth in HNSCC cells (Zheng et al., 2014). ROS can also induce tumor cells to secrete matrix metalloproteinases such as MMP-1, which promote vascular structure formation in the tumor microenvironment (Wartenberg et al., 2003).

Normal levels of ROS are important to T-cell activation and subsequent effects. For example, ROS regulate T cell activity by controlling IL-2 and IL-4 expression (Kaminski et al., 2010). Reducing T-cell-intrinsic ROS limits SENP7 cytosolic translocation and inhibits CD8^+^ T-cell metabolism and functional activity in human colorectal cancer cells (Wu et al., 2022). However, intolerable levels of ROS induced by mitochondrial dysfunction promote T-cell depletion, and lowering tumor hypoxia can limit T-cell exhaustion (Scharping et al., 2021). The redox status of T cells can also influence receptors on the surface of T cell membranes and thus affect signal transduction and distort immune responses (Kesarwani et al., 2013). Furthermore, immunosuppressive cells such as MDSCs increase ROS production in the tumor microenvironment, negatively affecting immune responses by stopping identification between the receptor TCR and MHC (Wei et al., 2015). T cells tend to become dysfunctional due to the immunosuppressive microenvironment. Hence, the treatment for regulating ROS or targeting its signaling may improve cancer immunotherapy. As a representative of stromal cells in the tumor microenvironment, cancer-associated fibroblasts (CAFs) are critical for increasing ROS levels in tumors (Chan et al., 2017).

**FIGURE 4:**
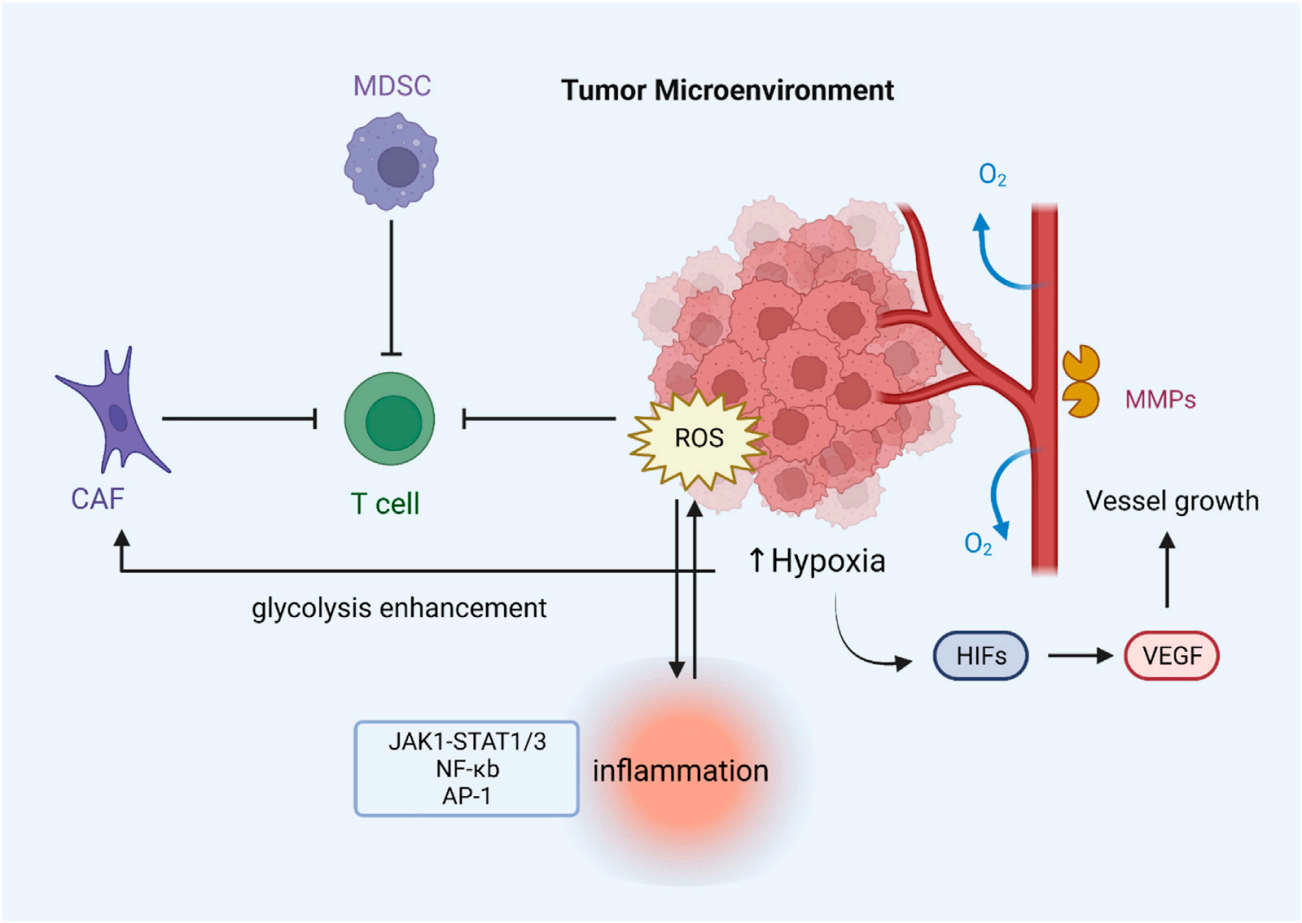
ROS regulate drug resistance by modulating the tumor microenvironment. Hypoxia can lead to ROS overproduction, which induces the expression of various angiogenic factors (VEGF) through the activation of HIF proteins, thereby promoting tumor angiogenesis. Increased release of MMP-1 contributes to the formation of vascular structures. MDSCs and CAFs can inhibit T-cell function by generating excessive ROS, thus leading to immune evasion of tumors. Increased ROS levels can also activate inflammation-related pathways and stimulate inflammasome formation.

On the other hand, ROS can reprogram the metabolic capacity of surrounding normal fibroblasts, turning them into the metabolic CAF phenotype in the TME (Zhang Z. et al., 2020). Oxidative ATM-mediated glycolysis enhancement in CAFs takes part in the metastasis of breast cancer cells (Sun et al., 2019). Another important feature of the tumor microenvironment is inflammation, which is closely related to the development of kinds of cancers, including gastric and nasopharyngeal cancer, and the efficacy of anticancer treatments (Zhao et al., 2021). Excessive ROS can promote the expression of proinflammatory cytokines by activating the transcription factors NF-KB and AP-1, which are sensitive to the redox state and may take part in activating the NLRP3 inflammasome (Hussain et al., 2016; Sho and Xu, 2019). Studies have shown that aloin alleviates LPS-induced inflammatory responses by inhibiting the ROS-mediated activation of the JAK1-STAT1/3 signaling pathway (Ma et al., 2018). In addition, ROS also form a regenerative feedback loop through autocrine TNF-α-mediated inflammatory cytokine/chemokine expression, which contributes to tumor progression (Blaser et al., 2016). In general, oxidative stress interacts with inflammation in the tumor microenvironment, and the specific mechanism still needs further investigation (Figure 4).

## 7 Clinical application of redox regulators

The increase of ROS in tumor cells is of great significance for drug therapy (Forman and Zhang, 2021; Ming et al., 2022). Moderate levels of ROS can promote tumor signal transduction, cell proliferation, gene susceptibility to mutation, and other factors contributing to cancer progression. However, raising intracellular ROS levels to toxicity thresholds can overcome cellular antioxidant defenses and kill cancer cells, thus providing opportunities for the development of drug-resistant cancer therapies. Conventional chemotherapy selectively kills cancer cells by inducing excessive oxidative stress (Table 1). Anthracycline drugs such as doxorubicin, daunorubicin, and adriamycin have been reported to have anticancer activity in solid and hematological cancers because they block DNA synthesis and induce ROS production (Young et al., 1981). In addition, studies have shown that combining chemotherapy with some natural products can boost anticancer effects (Table 2). For example, the synergistic impact of anethole with adriamycin can promote ROS-mediated apoptosis in triple-negative breast cancer cells (Arumugam et al., 2021). The combination of doxorubicin and oxymatrine exerted superior synergistic effects on colorectal cancer cells *in vitro* and *in vivo* than either DOX or OMT alone (Pan et al., 2021). Platinum-based drugs, such as cisplatin and carboplatin, are known to maintain very high ROS levels, inducing DNA damage and cancer cell death by promoting oxidative stress, especially the overproduction of mitochondrial ROS and the reduction of intracellular antioxidants such as glutathione in some human malignancies (Mikuła-Pietrasik et al., 2019). However, oxidative stress caused by cisplatin can cause common side effects in normal cells, such as ototoxicity, which is caused by the excess production of ROS in cochlear cells (Sheth et al., 2017). Moreover, paclitaxel, which inhibits cell mitosis in tumor therapy, has been found to promote ROS generation by enhancing the activity of NADPH oxidase (NOX) (Alexandre et al., 2007). ROS-based treatments vary widely depending on tumor type, location, and stage of cancer development. Therapies that are effective for one type of tumor may not be effective for another type of tumor, and inappropriate application of ROS modulation therapy may not only be ineffective, but may even promote malignant progression. Only more refined regulation is expected to overcome the controversial side effects of pro-oxidative antitumor therapy (Wang et al., 2021).

**TABLE 1:** Summary of redox sensors correlated with drug resistance.

Protein	Site	Modification	Function	Ref
MDR1	Cys431,Cys1074	-S-S-	Regulating drug transport activity	Urbatsch et al. (2001)
MRP1	MSD0	-S-S-	Regulating drug transport activity	Slot et al. (2011)
BCRP	Cys592,Cys603,Cys608	-S-S-	Regulating drug transport activity	Henriksen et al. (2005)
OGG1	Cys253,Cys255	Cys-S−	Inhibiting 8-oxOG damage repair	Bravard et al. (2006)
ERK1/2	Cys183	R-SNO	Decreasing the dual-phosphorylation of ERK1/2 on T183 and Y185	Jin et al. (2018)
ATG4B	Cys292,Cys361	-S-S-	Attenuating autophagic flux	Zheng et al. (2020)
LC3	Cys263,Cys572	-S-S-	Attenuating autophagic flux	Frudd et al. (2018)
PTEN	Cys124,Cys71	-S-S-	Activating the PI3K/AKT pathway	Leslie et al. (2003)
LAP-β	Met253	N.A.	Activating TGF-β1	Richter et al. (2015)
IKKβ	Cys179	S-glutathionylation	Inhibiting canonical NF-κB pathway	Reynaert et al. (2006)
p38α	Cys119,Cys162	-S-S-	Mediating the interaction between p38 and MKK3	Bassi et al. (2017)
CYPA	Cys115,Cys161	-S-S-	Buffering ROS	Peng et al. (2021)
PKM2	Cys358	N.A.	Inhibiting anaerobic glycolysis	Anastasiou et al. (2011)

N.A., not applicable.

**TABLE 2:** Summary of redox-regulated drugs under clinical evaluation.

Name	Mechanism	Cancer types	Effects on ROS	Clinical phase	Refs
Cisplatin	ROS-dependent DNA damage	Ovarian cancer	Induces a mitochondrial dependent ROS generation	Phase 2	NCT02608684
Doxorubicin	Induce cell apoptosis	Resistant solid malignancies	Increases ROS production	Phase 1	NCT00703170
Vorinostat	XCT inhibitor	Breast neoplasms	Reduces GSH	Phase 2	NCT02395627
Sulfasalazine	XCT inhibitor	Glioblastoma	Reduces GSH	Phase 1	NCT04205357
Sorafenib	XCT inhibitor	Ovarian cancer prostate cancer	Reduces GSH	Phase 2	NCT01047891
					NCT00414388
Paclitaxel	Inhibitor of cell division	Different types of cancer	Increases ROS production	Phase 3	NCT02728622
Carboplatin	ROS-dependent DNA damage	Prostate cancer	Increases ROS production	Phase 2	NCT00973882
Daunorubicin	ROS-dependent DNA damage	Leukemia	Increases ROS production	Not recruiting	NCT00898456
Disulfiram	NRF2 inhibitor	Prostate cancer	Increases ROS production	Phase 1	NCT02963051
Vitamin D	GPXs inhibitor	Colorectal cancer	Accumulate lipid peroxides	Phase 1	NCT05036109

Ferroptosis is a regulatory manner of non-apoptotic cell death promoted by increased lipid ROS, which is closely related to cysteine metabolism and oxidative stress (Dixon et al., 2012). In detail, inhibition of the xCT system on the cell membrane reduces intracellular glutathione synthesis precursors, thereby indirectly inhibiting glutathione peroxidase 4 (GPX4), ultimately leading to the accumulation of fatal lipid peroxides and ferroptosis (Imai et al., 2017). The lethal metabolic imbalance caused by glutathione depletion or inactivation of GPX4 is characteristic of ferroptosis (Friedmann Angeli et al., 2014). There is increasing evidence that ROS-induced ferroptosis helps to inhibit tumor growth and increase sensitivity to chemotherapy. NRF2 activation contributes to the resistance of head and neck cancer (HNC) cells to sorafenib, while inhibition of the NRF2-ARE pathway can induce ferroptosis and reverse chemotherapeutic drug resistance in HNC cells (Roh et al., 2017). The researchers found that cisplatin-resistant ovarian cancer cells pretreated with erastin (a ferroptosis inducer) showed increased sensitivity to cisplatin, demonstrating the good synergistic effect of the two drugs (Sato et al., 2018). In addition, inducing ferroptosis by erastin was found to reverse cisplatin resistance in HNC cells and significantly enhance the anticancer activity of the first-line chemotherapeutic agents cytarabine and doxorubicin in HL60 cells (Yu et al., 2015; Roh et al., 2016). Therefore, ferroptosis may play a key role in tumorigenesis, and drugs or molecules that induce ferroptosis can be used as adjuvant chemotherapy to treat drug-resistant tumors in clinical practice. In conclusion, a more profound perception of the role of oxidative stress and ferroptosis in tumors will create new opportunities for diagnosis and therapeutic interventions in drug-resistant tumors.

## 8 Conclusion and perspectives

The main obstacle to the efficient treatment of human malignant tumors is the emergence of multidrug resistance during chemotherapy. In this review, we summarized the most recent studies on ROS and its effect on cancer drug resistance, including drug transport and metabolism, DNA damage repair, downstream adaptive responses, stemness maintenance, and tumor microenvironment (Table 3).

**TABLE 3:** Summary of compounds that enhance the efficacy of classical chemotherapeutic drugs.

Compounds	Mode of action	Application	Tumor type	References
Apigenin	Increases ROS production	Increases the efficacy of 5-FU	hepatocellular carcinoma	Hu et al. (2015)
	Increases ROS production	Increases the efficacy of paclitaxel	HeLa cells	Xu et al. (2011)
Quercetin	Increases ROS production	Increases the efficacy of paclitaxel	Prostate cancer	Xiangyu Zhang et al. (2020)
Naringenin	Increases ROS production	Increases the efficacy of tamoxifen	Breast cancer	Xu et al. (2018)
Elesclomol	Impacts, ETC	Increases the efficacy of cisplatin	Melanoma	Cierlitza et al. (2015)
Rotenone	Impacts, ETC	Increases the efficacy of doxorubicin	Hepatocellular carcinoma	Li Wu et al. (2018)
Ampelopsin	upregulates NOX2 expression	Increases the efficacy of erlotinib	NSCLC	Hong et al. (2017)
Phenethyl isothiocyanate	Depletes GSH levels	Increases the efficacy of vorinostat	Leukemia	Hu et al. (2010)
	Depletes GSH levels	Increases the efficacy of Adramycin	Bladder carcinoma	Tang et al. (2013)
Cucurbitacin B	Increases ROS production	Increases the efficacy of cisplatin	Ovarian cancer	El-Senduny et al. (2016)
Hederagenin	Inhibits Nrf2 expression	Increases the efficacy of cisplatin	HNC tumors	Kim et al. (2017)
Salinomycin	Inhibits Nrf2 expression	Increases the efficacy of gefitinib	Nasopharyngeal carcinoma	Gong Zhang et al. (2017)

Moderate oxidative stress may induce antioxidant genes overexpression and ABC transporter synthesis in cells. However, severe oxidative stress may disrupt the transporter system and aid in overcoming drug resistance. Redox modification alters the conformation of drug efflux transporters by affecting intermolecular disulfide bonds, lowering drug efflux, and enhancing therapeutic effectiveness potential. ROS-induced DNA damage can cause early tumor generation, yet its DDR-inhibiting effect may help overcome tumor drug resistance. Furthermore, moderate ROS activate pro-survival signaling pathways (PI3K/AKT, MAPK, and NF-κB) as second messengers. The mutual regulation of ROS and autophagy also promotes tumor survival. However, under oxidative stress, excessive ROS may accelerate tumor cell apoptosis. ROS can also influence tumor energy metabolism by regulating critical metabolic enzymes, skewing the tumor toward the pentose phosphate pathway to maintain its reducibility. The early EMT driving process requires a high concentration of ROS. Still, only a tiny amount of ROS is needed to maintain tumor stem cell features, indicating that more sophisticated redox regulation therapy is required.

In the tumor microenvironment, hypoxia-induced ROS can promote vascular reconstruction and immune escape. Currently, given the complexity of the tumor microenvironment, the specific mechanism of tumor drug resistance is still relatively unknown. Meanwhile, as an important feature of the tumor microenvironment, inflammation is closely related to oxidative stress, which can also promote tumor drug resistance. In general, a considerable body of evidence suggests that tumor drug resistance is associated with frequent variations in the redox state, implying that redox signaling can be used to explain the molecular mechanism of tumor drug resistance and provide therapeutic methods.
